# The accuracy of different mismatch negativity amplitude representations in predicting the levels of consciousness in patients with disorders of consciousness

**DOI:** 10.3389/fnins.2023.1293798

**Published:** 2023-12-21

**Authors:** Kang Zhang, Kexin Li, Chunyun Zhang, Xiaodong Li, Shuai Han, Chuanxiang Lv, Jingwei Xie, Xiaoyu Xia, Li Bie, Yongkun Guo

**Affiliations:** ^1^Department of Neurosurgery, The First Hospital of Jilin University, Changchun, China; ^2^Department of Endocrinology, Jilin Province People’s Hospital, Changchun, China; ^3^Department of Neurosurgery, Qilu Hospital of Shandong University (Qingdao), Qingdao, China; ^4^Department of Neurosurgery, Siping Central People’s Hospital, Siping, China; ^5^Department of Neurosurgery, Fifth Affiliated Hospital of Zhengzhou University, Zhengzhou, China; ^6^Department of Neurosurgery, The First Medical Center of People’s Liberation Army (PLA) General Hospital, Beijing, China; ^7^Department of Neurosurgery, The Seventh Medical Center of Liberation Army (PLA) General Hospital, Beijing, China; ^8^Henan Key Laboratory of Brain Science and Brain Computer Interface Technology, Zhengzhou, China

**Keywords:** disorders of consciousness, mismatch negativity, microstate, functional connectivity, accuracy

## Abstract

**Introduction:**

The mismatch negativity (MMN) index has been used to evaluate consciousness levels in patients with disorders of consciousness (DoC). Indeed, MMN has been validated for the diagnosis of vegetative state/unresponsive wakefulness syndrome (*VS*/UWS) and minimally conscious state (MCS). In this study, we evaluated the accuracy of different MMN amplitude representations in predicting levels of consciousness.

**Methods:**

Task-state electroencephalography (EEG) data were obtained from 67 patients with DoC (35 *VS* and 32 MCS). We performed a microstate analysis of the task-state EEG and used four different representations (the peak amplitude of MMN at electrode Fz (Peak), the average amplitude within a time window −25– 25 ms entered on the latency of peak MMN component (Avg for peak ± 25 ms), the average amplitude of averaged difference wave for 100–250 ms (Avg for 100–250 ms), and the average amplitude difference between the standard stimulus (“S”) and the deviant stimulus (“D”) at the time corresponding to Microstate 1 (MS1) (Avg for MS1) of the MMN amplitude to predict the levels of consciousness.

**Results:**

The results showed that among the four microstates clustered, MS1 showed statistical significance in terms of time proportion during the 100–250 ms period. Our results confirmed the activation patterns of MMN through functional connectivity analysis. Among the four MMN amplitude representations, the microstate-based representation showed the highest accuracy in distinguishing different levels of consciousness in patients with DoC (AUC = 0.89).

**Conclusion:**

We discovered a prediction model based on microstate calculation of MMN amplitude can accurately distinguish between MCS and *VS* states. And the functional connection of the MS1 is consistent with the activation mode of MMN.

## 1 Introduction

Advancements in medical technology have enhanced the survival rate of patients with severe traumatic brain injury, leading to a growing number of patients with disorders of consciousness (DoC) (Wang et al., 2022), an unconscious state lasting ≥28 days, including vegetative state/unresponsive wakefulness syndrome (*VS*/UWS) and minimally conscious state (MCS) (Laureys et al., 2010; Bruno et al., 2011; Giacino et al., 2018). *VS*/UWS is defined as being awake but without behavioral signs of consciousness and is characterized by the absence of functional communication with the outside world but the presence of a sleep–wake cycle (Laureys et al., 2010). MCS is defined as having minimal, repetitive, but not sustained consciousness with the ability to perceive oneself and the external environment, as manifested by correct responses to verbal commands, object use, stimulus localization, and visual tracking, among others (Fins, 2002; Giacino et al., 2014). MCS is further divided into MCS+ and MCS-, where patients with MCS+ show compliance with commands and understandable or purposeful communication in language, while those with MCS- show compliance with commands but cannot achieve functional communication with the outside world (Thibaut et al., 2020).

The accurate assessment of the consciousness state of patients with DoC has important implications for guiding clinical decisions and predicting prognosis. Currently, the most clinically effective assessment tool is the JFK Coma Recovery Scale-Revised (CRS-R), which includes six subscales for auditory, visual, motor, verbal, communication, and arousal states, with a total score of 23 determined by the best score on each subscale (Giacino et al., 2004; Di et al., 2017). However, this diagnostic approach largely depends on the subjective interpretation of behavioral responses and cannot explore the “covert consciousness” of patients, such as those with cognitive motor dissociation (CMD) or locked-in syndrome (LIS) who may have perception and volitional thinking abilities but lack the ability for self-expression and motor output (Schiff, 2015; Edlow et al., 2021).

In addition to behavioral scales, neuroimaging (such as fMRI) and neurophysiological techniques (such as rest/task EEG and TMS-EEG) have been used to assess levels of consciousness in recent years. Although neuroimaging has a higher spatial resolution, it relies on hemodynamic responses and has a poor temporal resolution, whereas electroencephalography (EEG) has a high temporal resolution, is important for exploring instantaneous brain responses, and can serve as a bedside examination tool (Young et al., 2021). Neurophysiological measures have gradually evolved from the traditional standard EEG and somatosensory-evoked potentials to event-related potentials (ERP), quantitative EEG, and transcranial magnetic stimulation EEG (TMS-EEG) (Kondziella et al., 2020). Among these, auditory mismatch negativity (MMN) is an ERP that uses the oddball paradigm; a brief amplitude negative deflection is observed in neurophysiological recordings when a low-probability “deviant sound” occurs (Näätänen et al., 2004). Depending on the size of the deviant stimulus, this negative wave is observed at 100–250 ms post-stimulation. Furthermore, while MMN can be observed without conscious attention, its amplitude increases with enhanced consciousness (Alho, 1995). This indicates that patients with DoC can also develop MMN and be assessed for residual consciousness. Furthermore, DoC patients have shown significantly better clinical improvement with higher MMN amplitude and lower latency (Kotchoubey et al., 2005; Wijnen et al., 2007). Therefore, MMN is a potential index to detect the residual information-processing ability of patients with DoC (Fischer et al., 2010).

The identification of ERP components is primarily based on their corresponding latencies and amplitudes within specific time windows; for example, MMN between 100 and 250 ms, in which P300 usually appears around 300 ms after stimulus onset (Tommaso et al., 2020).

Furthermore, ERP components are mainly distributed in specific regions, with MMN mainly detected in the frontal-central region of the scalp (Fitzgerald and Todd, 2020). However, owing to the heterogeneity of etiology in patients with DoC, obtaining MMN based on traditional methods may not correspond to the patient’s actual residual consciousness.

However, EEG microstates are used to study rapid changes in global brain states. These are defined as semi-stable configurations of scalp potential fields that appear as repetitive sequences over consecutive short time intervals (Khanna et al., 2015; Michel and Koenig, 2018). Microstate configurations range from 4 to 7 classically distinct topographical patterns explaining at least 70% of the recorded data (Khanna et al., 2015). Studies on healthy subjects classified microstates into four types and their corresponding resting-state brain networks were identified through EEG-functional magnetic resonance imaging (fMRI) integration (Khanna et al., 2015). Most studies have focused on the microstate analysis of resting-state EEG to improve the diagnosis of mental and neurological disorders (Schumacher et al., 2019; Da Cruz et al., 2020; Hao et al., 2022). Although Gui et al. (2020) achieved good predictive results for the prognosis of patients with DoC by combining language stimulation paradigms with microstate analysis (Gui et al., 2020), data on DoC based on microstates, particularly in the context of ERP analysis, remain limited. Especially, research on MMN in patients with DoC lacks spatiotemporal EEG analysis of response-locked MMN. In addition, ERP components are manifestations of specific cognitive processes at the brain level, with the physical properties of different components (such as latency and amplitude) reflecting different aspects of cognitive processes (Näätänen et al., 2004).

This study analyzed the microstates of task-related EEG in patients with DoC, identified the microstate categories that induced MMN, and conducted functional connectivity analysis of this category to determine the MMN functional connectivity patterns. Furthermore, we proposed a novel method for identifying MMN and identified the MMN threshold to clarify the level of consciousness.

## 2 Materials and methods

### 2.1 Participants

From January 2018 to August 2022, we recruited 67 patients with DoC (35 *VS* and 32 MCS) from the Fifth Affiliated Hospital of Zhengzhou University and Zhengzhou Central Hospital (Zhengzhou University) Neurosurgery Department. The patient age range was 18–82 years (mean, 52.8 ± 15.2) with a mean illness duration of 1.5–24 months (mean, 3.23 ± 4.38). The etiologies included traumatic brain injury (22 cases), ischemic hypoxic encephalopathy (9 cases), and stroke (36 cases). The age and disease duration did not differ significantly between the *VS* and MCS groups (*p* > 0.05). Experienced clinicians used the CRS-R to evaluate each participant (Giacino et al., 2004).

The inclusion criteria were: (1) *VS*/UVS or MCS diagnosis based on CRS-R score and disease duration ≥28 days; (2) intact skull without intracranial implants; (3) presence of a sleep–wake cycle; (4) autonomous breathing and stable blood pressure; (5) spontaneous eye opening or in response to stimulation; (6) not administered anesthesia or sedatives within 48 h before monitoring; (7) signed informed consent.

The exclusion criteria were: (1) disease duration <28 days; (2) presence of a sleep–wake cycle but an inability to spontaneously open eyes; (3) skull damage or loss; (4) intracranial implants; (5) presence of severe life-threatening complications (pulmonary infection, shock, and seizures); (6) administration of anesthesia or sedatives within 48 h before monitoring.

Written informed consent was obtained from all the patients’ family members and caregivers. This study was conducted in accordance with the principles outlined in the Declaration of Helsinki and was approved by the Ethics Committee of the Fifth Affiliated Hospital of Zhengzhou University (approval number: KY2020024) and Zhengzhou Central Hospital affiliated with Zhengzhou University (approval number: 201614).

### 2.2 Methods

#### 2.2.1 Mismatch negativity paradigm

The pure-tone oddball paradigm was used to elicit MMN, with a 1,000 Hz pure tone as the standard stimulus (“S”), and a 1,200 Hz pure tone as the deviant stimulus (“D”). The paradigm comprised 1,000 sound stimuli lasting for 200 ms, with stimuli onset not synchronized at 1,000 ms. The stimuli were uninterrupted and pseudorandomly presented, with probabilities of 0.8 and 0.2 for standard and deviant stimuli, respectively. At least three standard stimuli were presented between two consecutive deviants. The stimulus sequence was programmed using E-Prime version 3.0 (Psychology Software Tools, Pittsburgh, PA, United States) and delivered through headphones. The experiment lasted approximately 17 min.

#### 2.2.2 Event-related potentials data acquisition and processing

Prior to EEG recording, the CRS-R arousal protocol was used to awaken the patient and maintain wakefulness during EEG acquisition. A Nicolet amplifier (Natus Neurology Corporation) was used to record scalp EEG data from 28 electrodes, according to the 10/20 international system. The sampling rate was 1,000 Hz and the electrode impedance was <5 K. Offline preprocessing was performed using EEGLAB and custom scripts (Delorme and Makeig, 2004) in MATLAB (MathWorks, Natick, MA, United States). First, the EEG data were bandpass-filtered (1–30 Hz), and a notch filter (48–52 Hz) was applied to remove the AC influence (50 Hz IF signal). The sampling rate was then down-sampled to 500 Hz and independent component analysis (ICA) was applied to filter out blinks, horizontal eye movements, muscle activity, and electrocardiogram artifacts in the spatial domain. EEG data with wave amplitudes between −100 and 100 μV were retained. Finally, > 80% of the data were retained for each patient.

#### 2.2.3 Extracting epochs and averaging and calculating the difference waves

Preprocessed EEG data were segmented into 800 ms epochs (stimulus onset: 0 ms, baseline period: −200–0 ms). The baseline was subtracted from each trial to ensure that all ERP segments had the same origin. The S and D segment events were separately averaged to obtain the standard and deviant stimulus waveforms, and the difference was calculated. MMN was defined as the maximum negative wave within 100–250 ms. The Fz electrode was defined as the region of interest.

#### 2.2.4 Criteria for identifying and quantifying MMN properties

The standard for identifying MMN components was as follows: (1) the presence of N100 as a prerequisite for measuring the MMN component; (2) identifying the most negative peak (peak of the MMN component) within 100–250 ms at electrode Fz, with the corresponding time latency of the MMN component; (3) calculating the peak amplitude of MMN at electrode Fz(Peak), the average amplitude within a time window −25–25 ms entered on the latency of peak MMN component(Avg for peak ±25 ms), the average amplitude of averaged difference wave for 100–250 ms (Avg for 100–250 ms), and the average amplitude difference between the standard stimulus (“S”) and the deviant stimulus (“D”) at the time corresponding to Microstate n(MS n) (Avg for MS n). MS n represented one of the cluster maps.

#### 2.2.5 Microstate-based analysis

ERP microstate analysis was performed using CARTOOL (Brunet et al., 2011). First, the grand average ERP was calculated for both conditions, followed by topographic atomization and an agglomerate hierarchical clustering (T-AAHC) to segment the grand average ERP in both time and space. The hierarchical clustering approach operates in a bottom-up manner wherein the number of clusters is initially large and progressively diminishes (Murray et al., 2008). Briefly, all maps submitted to the procedure (CARTOOL) are initially considered to be independent clusters. In each iteration of the algorithm, the “worst” cluster is identified and split into its constituent maps (“atomized”). The “worst” cluster in each iteration is the one with the lowest summated correlation between each constituent map to the average cluster map. Maps of the “worst” cluster are redistributed (“agglomerated”) to any of the remaining clusters to which they are most strongly correlated. This process is continued until the desired number of clusters is achieved (Khanna et al., 2014). The range of clusters was 1–20, while the correlation between the original and template topographic maps at each time point was >50%. Microstates with >95% similarity were combined into one class, while those with a duration of <6 ms were smoothed and assigned to other microstates. Furthermore, other parameters were included; for example, the window half size of temporal smoothing was 3 with a strength of 10; the temporal threshold of the rejected segment was 3 Time Frames (TFs; one TF is equivalent to 2 ms). The polarity of the brain topography map was considered in the ERP microstate analysis. The optimal cluster number was determined based on the global variance explained (GEV) and meta-criterion (MetaCrit) (Bréchet et al., 2019). To determine the optimal number of clusters, six criteria, namely Gamma, Silhouettes, Davies and Bouldin, Point-Biserial, Dunn, and Krzanowski-Lai Index were used to independently evaluate the quality of each clustering and merged to derive a single synthetic MetaCrit. This improves the confidence in the estimation of the optimal number of clusters compared with previous work relying on a single criterion only (Pascual-Marqui et al., 1995; Murray et al., 2008).

For back-fitting, the group cluster maps of the optimal cluster number were fitted to the average EEG data of S and D conditions separately for each subject, including all averaged data points of each subject (not only GFP peaks). The polarity of the maps was considered in this back-fitting procedure. Data points where none of the cluster maps reached a correlation higher than 50% were labeled as “non-assigned.” Once the whole recording was labeled, temporal smoothing was applied by ignoring segments where a given cluster map was present for less than 20 ms, and the time points were split and assigned to the preceding and following cluster maps. The parameter was quantified for each subject, respectively the cluster map(microstate) of each data point, as well as the duration of each cluster map.

#### 2.2.6 Microstate-wise functional connectivity

The functional connectivity of each microstate category was computed for all channel pairs in the alpha (8–13 Hz) ranges using the phase-locking value (PLV) method based on phase synchronization. The CSD toolbox (Kayser and Tenke, 2006) was used to apply a surface Laplacian transformation to the EEG data to overcome volume conduction issues. Next, after filtering the data into a narrow band (alpha 8–13 Hz), the Hilbert transform was calculated. Data segments belonging to specific microstate categories were selected and concatenated. Subsequently, the samples were epoched into non-overlapping 4-s windows, and phase synchronization was calculated using the PLV. This method was repeated for each microstate class to obtain functional connectivity patterns.

### 2.3 Statistical analysis

Statistical analyzes were performed using R software (The R Project for Statistical Computing, Vienna, Austria). Among demographic variables, chi-square tests were used for sex and etiology, while *t*-tests were used for post-injury duration, age, and CRS-R total scores. Shapiro–Wilk tests were performed to analyze distributional characteristics. Non-parametric Kruskal–Wallis tests were used to compare the duration of each microstate in the 100–250 ms time window, including the duration of each microstate between conditions “S” and “D” and the duration of each microstate between MCS and *VS* groups. The Pairwise Wilcoxon Rank Sum Test was used to calculate pairwise comparisons between group levels of microstate category with Bonferroni corrections for multiple testing. We determined the most statistically significant microstate category (“MS n”) using post-hoc tests.

Wilcoxon Signed Rank Test were used to analyze the statistical differences in Peak, Avg for peak ±25 ms, Avg for 100–250 ms, and Avg for MS n. Bonferroni correction was used to control for the multiple comparisons among the four MMN amplitude representation modes. The *t*-tests passed the normality and homogeneity of variance tests.

Receiver operating characteristic (ROC) curves are useful tools to evaluate classifiers in biomedical applications. A ROC plot displays the performance of a binary classification method with continuous or discrete ordinal output. In the ROC context, the area under the curve (AUC) measures the performance of a classifier and is frequently applied for method comparison. A higher AUC indicates better classification; therefore, to evaluate the accuracy of different MMN representations for diagnosing *VS* or MCS in patients with DoC, we performed ROC curve analysis on four amplitude representations and calculated the classification performance of AUC evaluation. Moreover, the cutoff value was determined to quantify the classification effect.

## 3 Results

### 3.1 Epidemiological characteristics

Of the 79 patients with DoC who were initially included, 67 met the inclusion criteria. There were 24 female participants in the MCS group and 11 in the *VS* group. The sex distribution did not differ between the MCS and *VS* groups as determined by the chi-square test (*p* = 1.00). Twelve patients were excluded due to skull defects, epileptic seizures, or sedative use. The mean patient ages were 53.8 ± 15.2 and 51.8 ± 15.7 years for the MCS and *VS* groups, respectively (*p* = 0.589). The MCS group included 2 cases of hypoxic–ischemic encephalopathy, 20 cases of stroke, and 13 cases of traumatic brain injury, compared with 6, 17, and 9 cases, respectively, in the *VS* group (*p* = 0.241). The average durations after injury were 2.74 ± 3.98 and 3.81 ± 4.77 months for the MCS and *VS* groups, respectively (*p* = 0.325). The total CRS-R scores were 10.8 ± 3.02 and 4.88 ± 1.44 for the MCS and *VS* groups, respectively (*p* < 0.001). The epidemiological characteristics of the patients are presented in Table 1.

**Table 1:** Patient characteristics by groups.

	MCS (*N* = 35)	*VS* (*N* = 32)	*p*-value
Gender			1.000
F	11 (31.4%)	11 (34.4%)	
M	24 (68.6%)	21 (65.6%)	
Age			0.589
Mean (SD)	53.8 (15.2)	51.8 (15.7)	
Median [Min, Max]	54.0 [18.0, 82.0]	54.5 [18.0, 80.0]	
Post-injury			0.325
Mean (SD)	2.74 (3.98)	3.81 (4.77)	
Median [Min, Max]	2.00 [1.00, 24.0]	2.00 [1.00, 24.0]	
Etiology			0.241
Anoxia	2 (5.7%)	6 (18.8%)	
Stroke	20 (57.1%)	17 (53.1%)	
TBI	13 (37.1%)	9 (28.1%)	
CRS-R			<0.001
Mean (SD)	10.8 (3.02)	4.88 (1.41)	
Median [Min, Max]	10.0 [6.00, 17.0]	5.00 [2.00, 9.00]	

MCS, minimally conscious state; VS, vegetative state; F, female; M, male; TBI, traumatic brain injury; CRS-R, coma recovery scale-revised; SD, standard deviation.

### 3.2 Microstate analysis

**Figure 1:**
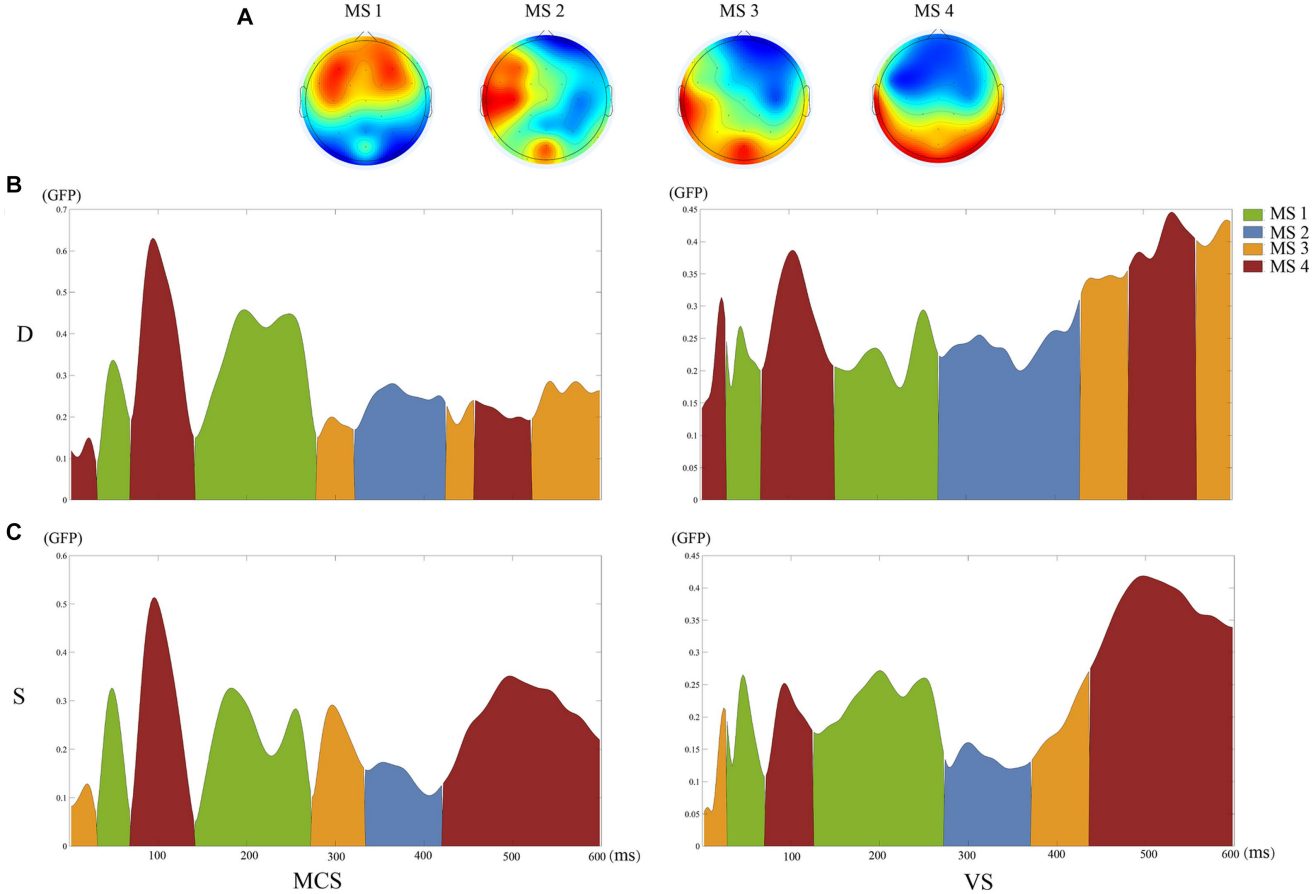
Results of the microstate analysis. **(A)** The topographic maps of the four clustered microstates. **(B)** The time distributions of the topographic maps for the MCS and *VS* groups under D conditions through spatiotemporal segmentation analysis. **(C)** The time distributions of the topographic maps for the MCS and *VS* groups under S conditions through spatiotemporal segmentation analysis. Different colors of the GFP curve represent the four microstate classes. GFP, Global Field Power.

The clustering of the four microstates showed a GEV of 89.61%. Figure 1A shows the topographic maps of the four clustered microstates. Figures 1B,C shows the time distributions of the topographic maps for the MCS and *VS* group under S and D conditions through spatiotemporal segmentation analysis. The duration of the four microstates differed significantly for 100–250 ms (Kruskal–Wallis, *p* < 0.01). Post-hoc analysis showed that MS 1 was significant in terms of time proportion during this time interval (Table 2).

**Table 2:** Results of statistical testing for the duration of four microstates.

Groups	D	S
MS1-MS2	<0.001	<0.001
MS1-MS3	<0.001	<0.001
MS1-MS4	<0.001	<0.001
MS2-MS3	0.06	1.00
MS2-MS4	1.00	1.00
MS3-MS4	0.05	0.23

MS1, MS2, MS3, and MS4 refer to four microstates respectively; D and S refer to condition D and condition S, respectively.

In Supplementary Table S1, the mean duration of MS1 were 86.71 ± 15.50 ms for condition “D” and 71.16 ± 7.95 ms for condition “S” (*p* = 0.10). For MS2, the mean duration was 16.93 ± 10.75 ms for condition “D” and 10.45 ± 5.29 ms for condition “S” (*p* = 0.15). For MS3, the mean duration was 7.49 ± 4.72 ms for condition “D” and 7.34 ± 5.09 ms for condition “S” (*p* = 1.00). For MS4, the mean duration was 19.34 ± 4.30 ms for condition “D” and 13.04 ± 6.66 ms for condition “S” (*p* = 0.18).

In Supplementary Table S2, the mean duration of MS1 were 65.43 ± 14.25 ms for MCS group and 63.91 ± 15.35 ms for *VS* group (*p* = 0.87). For MS2, the mean duration was 14.05 ± 8.62 ms for MCS group and 13.28 ± 8.02 ms for *VS* group (*p* = 0.93). For MS3, the mean duration was 9.16 ± 5.85 ms for MCS group and 5.83 ± 4.90 ms for *VS* group (*p* = 0.20). For MS4, the mean duration was 16.68 ± 13.45 ms for MCS group and 15.66 ± 11.32 ms for *VS* group (*p* = 0.99).

### 3.3 Mismatch negativity amplitude

**Figure 2:**
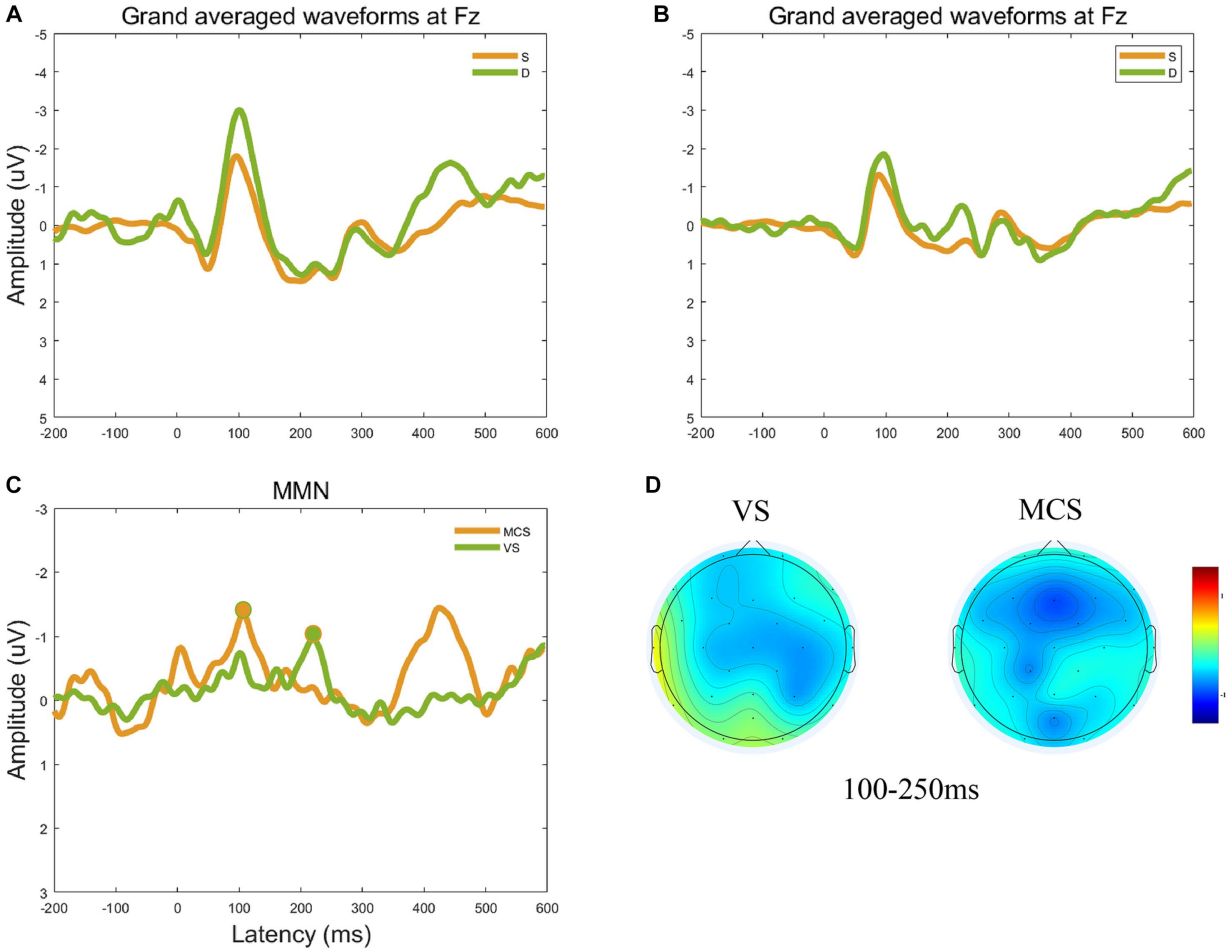
Group-averaged ERP and MMN waveforms at the electrode Fz. **(A)** The group-averaged ERP waveforms at the Fz electrode for the MCS group. **(B)** The group-averaged ERP waveforms at the Fz electrode for the *VS* group. **(C)** Group-averaged MMN waveforms at electrode Fz and obvious MMN components peak in the circular shadow area. The peak amplitude of group-averaged MMN measured −1.34 μV with a latency of 106 ms in the MCS group, and − 0.95 μV with a latency of 220 ms in the *VS* group. **(D)** The topographic changes in average MMN waveforms from 100 to 250 ms for the *VS* and MCS groups.

This study focused on the auditory evoked potential MMN. The Fz electrode on the frontocentral scalp was selected as the observation electrode since MMN mainly occurs in the frontal region. The group-averaged ERP waveforms at the Fz electrode are shown in Figures 2A,B. The group-averaged MMN measured at −1.34 μV with a latency of 106 ms in the MCS group, and − 0.95 μV with a latency of 220 ms in the *VS* group (Figure 2C). Figure 2D shows the topographic changes in average MMN waveforms from 100 to 250 ms. In the frontal region, the amplitude of the MMN waveform was significantly higher in the MCS than *VS* groups.

**Figure 3:**
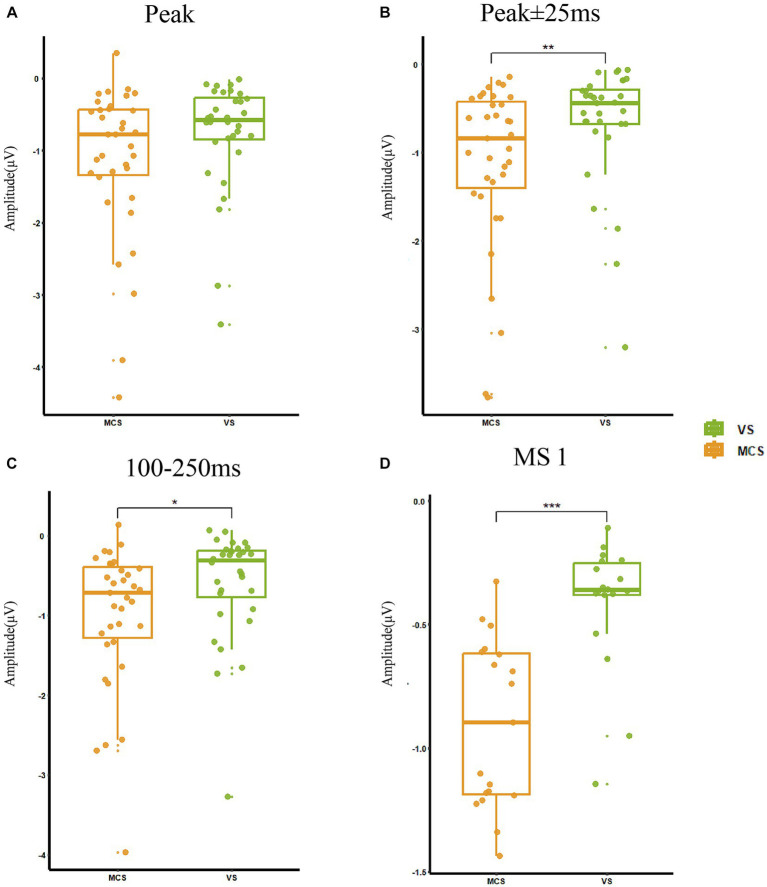
MMN representations in MCS and *VS* groups. **(A)** The peak amplitude was −1.06 ± 0.40 μV and there was no significant change between the two groups (*p* = 0.07). **(B)** The average amplitude in the 25 ms window before and after the peak was −0.98 ± 1.06 μV, and MCS had a significantly increased amplitude (*p* < 0.01) compared with VS. **(C)** The average amplitude at 100–250 ms was −0.75 ± 1.00 μV, and MCS had a significantly increased amplitude (*p* = 0.01) compared with VS. **(D)** The average amplitude difference between stimulus conditions S and D at the time corresponding to MS 1 was −0.69 ± 0.40 μV, and MCS had a significantly increased amplitude (*p* < 0.001) compared with VS. *p < 0.05, **p < 0.01, ***p < 0.001.

The statistical indicators used in this study were the peak amplitude of MMN at electrode Fz(Peak), the average amplitude within a time window −25–25 ms on the latency of peak MMN component (Avg for peak ±25 ms), the average amplitude of averaged difference wave for 100–250 ms (Avg for 100–250 ms), and the average amplitude difference between stimulus conditions S and D at the time corresponding to MS 1 (Avg for MS1). In MCS group, the Peak was −1.33 ± 1.42 μV, the Avg for peak ±25 ms was −1.27 ± 1.26 μV, the Avg for 100–250 ms was −1.00 ± 0.88 μV, and the Avg for MS1 was −0.89 ± 0.37 μV. In *VS* group, the Peak was −0.77 ± 0.77 μV, the Avg for peak ±25 ms was −0.66 ± 0.69 μV, the Avg for 100–250 ms was −0.48 ± 1.06 μV, and the Avg for MS1 was −0.44 ± 0.29 μV. Evaluation of the differences in MMN representations between the MCS and *VS* groups showed significantly higher average amplitudes in the 25 ms window before and after the peak (*p* < 0.01), at 100–250 ms (*p* = 0.01), and the average amplitude difference between stimulus conditions S and D at MS 1 (*p* < 0.001) in the MCS group compared with the *VS* group. The peak amplitude (*p* = 0.07) was also higher in the MCS group. The above results are shown in Figure 3.

### 3.4 Microstate functional connectivity

**Figure 4:**
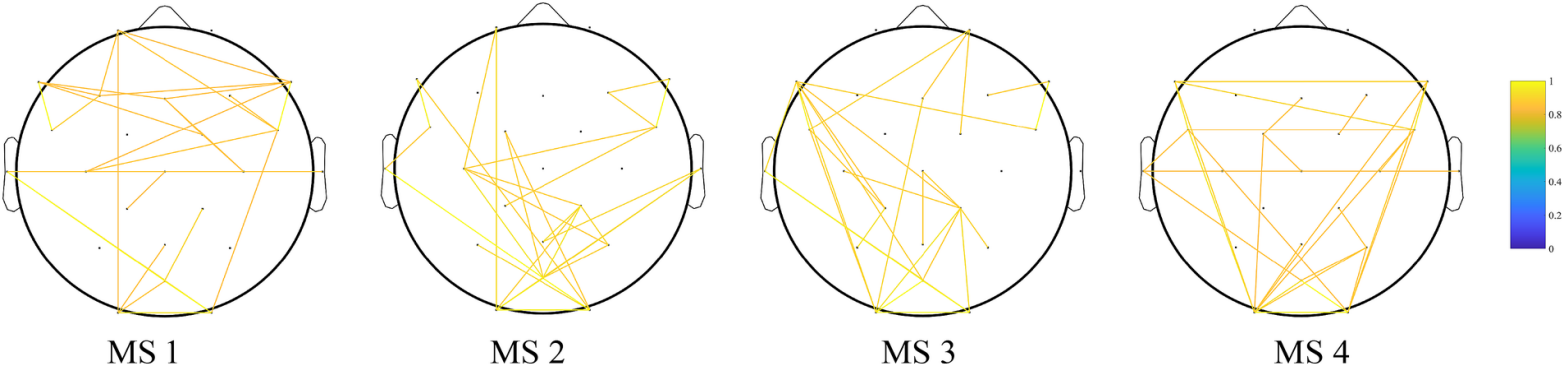
Results of the PLV functional connectivity analysis of MS 1, MS 2, MS 3, and MS 4. Edges show the top 1% of edges that deviate from static background connectivity. The frontal lobe mainly showed increased functional connectivity in MS 1.

MS 1 was likely associated with brain network changes induced by MMN in response to auditory stimuli. Figure 4 shows the results of the PLV functional connectivity analysis of MS 1, MS 2, MS 3, and MS 4. The frontal lobe mainly showed increased functional connectivity in MS 1.

### 3.5 Diagnostic prediction

**Figure 5:**
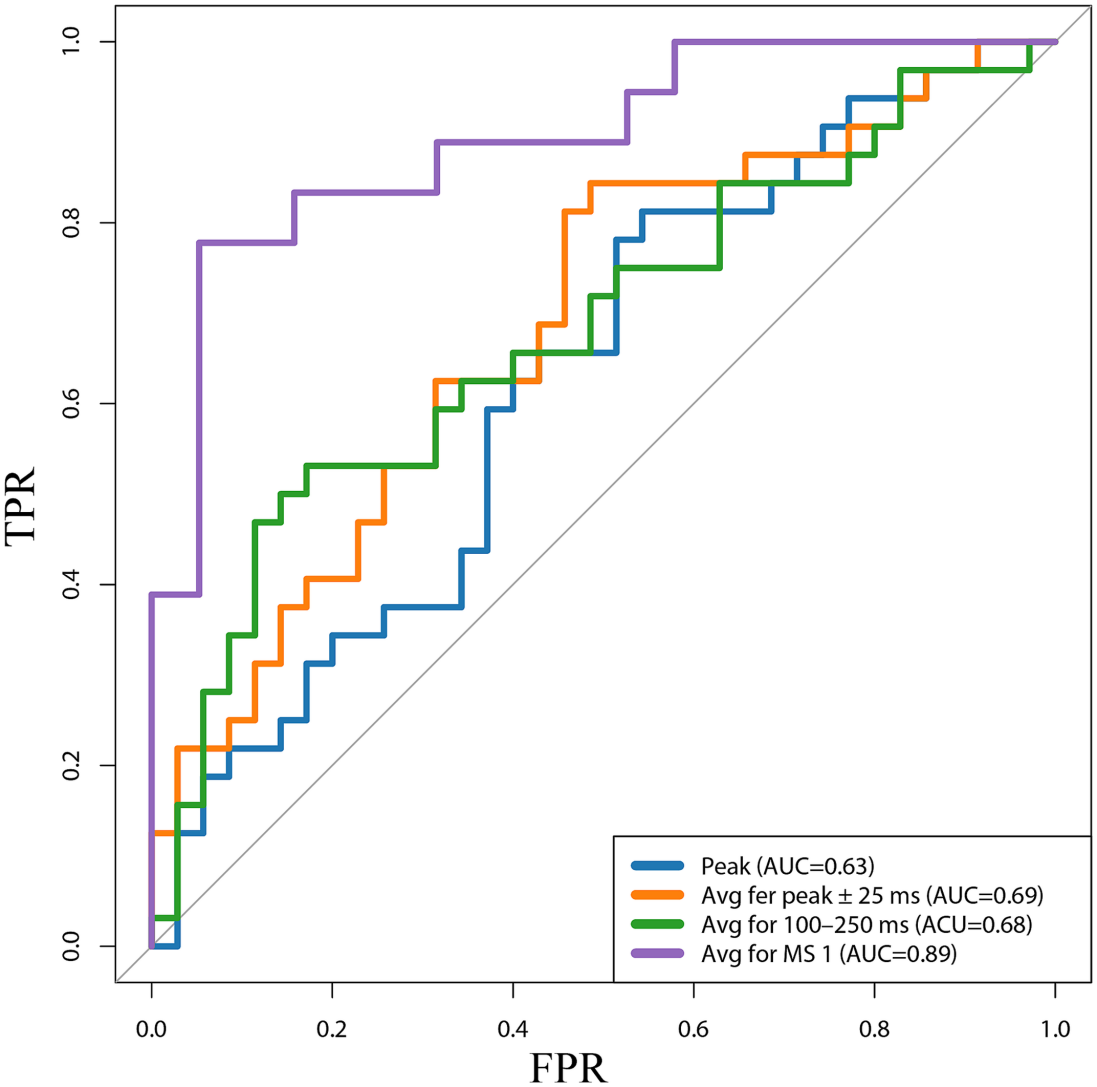
Receiver operating characteristic (ROC) curves for the four different methods of representing MMN amplitudes. Peak, the peak amplitude; Avg fer peak ± 25 ms, the average amplitude in the 25 ms window before and after the peak; 100–250 ms, Avg for 100–250 ms, the average amplitude at 100–250 ms; Avg for MS 1, the average amplitude difference between stimulus conditions S and D at the time corresponding to MS 1; TPR, true positive rate; FPR, false positive rate; AUC, the area under the curve.

We plotted the ROC curves for the four different methods of representing MMN amplitudes (Figure 5). The horizontal and vertical axes represent the false positive rate (FPR) and true positive rate (TPR), respectively. MS 1 showed a good upward trend in the ROC curve of the model, representing a difference in mean amplitude between condition “D” and “S,” and was significantly higher than the random level. The AUC was 0.89, suggesting that our model correctly classified the probability of the true label being higher than that of random guessing by 89%. Compared with the ROC curves and AUC values of the other three MMN representation methods (Peak AUC = 0.63, Avg for peak ±25 ms AUC = 0.69, and Avg for 100–250 ms AUC = 0.68), Avg for MS1(AUC = 0.98), showed excellent predictive accuracy. We determined the optimal classification threshold (cutoff value) for the final classification result. The cutoff value for the Peak model was −1.05 μV, with a true positive rate of 0.81 and a false positive rate of 0.54. That is, MCS is diagnosed when the predicted value of the model is ≤ −1.05 μV. The cutoff value for the model corresponding to Avg for MS1 was −0.43 μV. Although this cutoff differed from those of traditional MMN calculation methods, its predictive performance was good.

## 4 Discussion

The brain is a complex and dynamic information-processing organ with stable structural and functional connections between regions for the transmission of temporal information (Park et al., 2021). Patients with DoC have varying degrees and causes of brain damage, resulting in a reduced capacity to process external information. Recently, significant progress has been made in investigating true remaining consciousness in patients with DoC. MMN is a product of conscious brain activity generated when the brain detects deviant stimuli and reflects a relatively intact brain structure and function (Fitzgerald and Todd, 2020). In patients with DoC, MMN indicates better underlying cognitive function and can serve as an indicator of consciousness and prognostic prediction. Previous studies comparing MMN amplitudes and latencies in healthy controls, patients in MCS, and patients in *VS* as references demonstrated the importance of MMN in DoC.

Boly et al. (2011) induced MMN waveforms in 21 patients with DoC and 21 healthy controls and observed a stronger reaction in the control than DoC groups (Boly et al., 2011). Wang et al. (2017) reported that sudden increases in MMN amplitude and shortening of latency may indicate improved consciousness in patients with DoC (Wang et al., 2017). Höller et al. (2011) evaluated MMN in 6 patients with MCS, 16 with *VS*, and 15 healthy controls, reporting significant MMN in only 73% of healthy controls and 12.5% of *VS* patients (Höller et al., 2011). In our study, the MCS group had a peak amplitude of −1.34 μV at 100–250 ms, which was higher in absolute value than that of the *VS* group (−0.95 μV), indicating that our results are consistent with previous research; namely, the higher the level of consciousness, the stronger the patient response to deviant stimuli.

Previous studies have determined the MMN amplitude and latency by determining the most negative point within the 100–250 ms window at the frontocentral site. However, the quality of EEG data acquisition, level of post-analysis processing, patient state, and the surrounding environment affect MMN accuracy. Therefore, various approaches have been explored to improve the accuracy of MMN representations, including source localization of the induced MMN (Tsolaki et al., 2015, 2017; Wang et al., 2017). We previously identified MMN based on the most negative peak (Wang et al., 2020).

In the microstate analysis, each microstate category represents an ERP component. Laganaro (2017) applied microstate analysis to data from 118 subjects administered facial stimuli from different datasets and found that different subjects produced the same microstate sequence in the same time series, thereby demonstrating the accuracy of this approach (Laganaro, 2017). From previous research on resting-state microstates, each category represents a brain network activation pattern (Zhang et al., 2023). In the MCS and *VS* groups in the present study, MS 1 showed the most difference between 150 and 250 ms, suggesting that it represents the MMN activity pattern. Therefore, MS 1 likely represented the main activity pattern of the brain in patients with DoC receiving deviant auditory stimuli.

To demonstrate the accuracy of our hypothesis, we conducted a functional connectivity analysis of four maps of microstate analysis in the alpha (8–13 Hz) ranges. The current prevailing theories regarding the mechanism of MMN are the “memory mismatch” and “deviance detection” hypotheses, which suggest that MMN is caused by a neural mismatch between standard and deviant stimuli in the sensory memory trace (Näätänen, 1995; Fitzgerald and Todd, 2020). This automatic mismatch process plays a critical role in detecting auditory changes outside the realm of attention. Additionally, a key feature of MMN is that it can be observed in patients who are unaware of the sound stream, under passive listening conditions, or with decreased consciousness, such as coma and sleep, indicating that complex sensory discrimination processes are initiated at a pre-attentive level (Garrido et al., 2009). Näätänen et al. (2014) demonstrated that brain regions predominantly activated by MMN are located in the frontal lobes, Opitz et al. (2002) confirmed this finding by combining MMN with functional magnetic resonance imaging (fMRI). Therefore, our functional connectivity analysis of microstate showed that the areas of functional connectivity were primarily located in the frontal lobe agrees, similar to previous MMN studies. Therefore, MS 1 represents the activity pattern of MMN in patients with DoC.

Our results demonstrated the categorization of MMN brain activity into microstates. To further explore the potential of MMN expression as an electroencephalographic biomarker for the diagnosis of patients with DoC, we compared four methods for determining the MMN, namely Peak, Avg for peak ±25 ms, Avg for 100–250 ms, and Avg for MS1. All methods demonstrated higher reactivity in the MCS then *VS* groups, and therefore can be used to assess consciousness level.

ROC curve analysis was performed to explore the optimal MMN amplitude representation approach. Analysis of the MMN amplitudes determined by these four methods identified MMN cutoff values, represented by Peak and Avg for peak ±25 ms, as diagnostic tools for MCS and *VS* of −1.05 μV and − 0.84 μV, respectively. These values were consistent with those of the MMN longitudinal study by Wijnen et al. (2007). Although the optimal diagnostic thresholds obtained by these two methods were similar to those in previous studies, their classification accuracies were not high. In contrast, Avg for MS1 showed an AUC of 0.89; therefore, we concluded that this new method of expressing MMN was more valuable for the diagnosis and prediction of patients with DoC. We also calculated a cutoff value of this new method of −0.43 μV. However, there remain significant differences between the use of microstates to express MMN and traditional peak values. This difference may be since the result is the average of relatively long-term amplitudes. Further research is required to determine the underlying reasons.

We conducted ROC analysis based on the four methods of calculating MMN amplitude and showed that the Avg for MS1 had a higher accuracy for the diagnosis of *VS* and MCS (AUC = 0.89). Our study differs from most prediction models for patients with DoC; moreover, we described a more accurate and objective method to demonstrate MMN significance.

This study has some limitations. First, we did not include a healthy control group, and our findings may only reflect the activation of MMN in the brain during DoC and not be applicable to all cases. Additionally, we did not evaluate medication and etiology as covariates that may affect changes in electroencephalogram indicators. Second, the small sample size included in this study (22 TBI cases, 9 hypoxic–ischemic encephalopathy cases, and 36 stroke cases) and the imbalance between the etiologies reduced the validity of our results were also major limitations of this study. Our data only recorded 28 electrodes, which might have influenced the results. Furthermore, this study lacked follow-up evaluations to verify the accuracy of the diagnostic predictions. Although electroencephalography is a functional neuroimaging tool, some results may be limited by functional connections and/or metabolic influences within brain regions. Therefore, multimodal techniques (fMRI and PET) remain warranted to further explore the underlying mechanisms, establish more typical multidimensional quantitative indicators, and detect brain imaging biomarkers.

## 5 Conclusion

In this study, we proposed a novel approach to interpret MMN components using the microstate method, demonstrated MMN activation patterns using functional connectivity analysis, and developed a predictive model based on the microstate-calculated MMN amplitude, which accurately distinguished the MCS and *VS*/UWS states.

## Data availability statement

The protocol and the statistical analysis plan will be made available by the corresponding author upon reasonable request. Due to the privacy regulations of the collaborating hospital, the individual EEG can only be accessible by contacting YG with a research application.

## Ethics statement

The studies involving humans were approved by this study was conducted in accordance with the principles outlined in the Declaration of Helsinki and was approved by the Ethics Committee of the Fifth Affiliated Hospital of Zhengzhou University and Zhengzhou Central Hospital affiliated with Zhengzhou University. The studies were conducted in accordance with the local legislation and institutional requirements. The participants provided their written informed consent to participate in this study.

## Author contributions

KZ: Formal analysis, Methodology, Writing – Original draft. KL: Data curation, Writing – review & editing. CZ: Methodology, Writing – Original draft. XL: Formal analysis, Writing – Original draft. SH: Data curation, Writing – Original draft. CL: Formal analysis, Writing – Original draft. JX: Formal analysis, Writing – Original draft. XX: Investigation, Writing – Original draft. LB: Writing – review & editing. YG: Data curation, Formal analysis, Writing – Original draft.
