# Supplementary material for: The accuracy of different mismatch negativity amplitude representations in predicting the levels of consciousness in patients with disorders of consciousness
Source: Front Neurosci. 2023 Dec 21;17:1293798. doi: 10.3389/fnins.2023.1293798 (PMC10764429; doi:10.3389/fnins.2023.1293798)
Supplement: Supplementary file 1 [file Data_Sheet_1.PDF]

The Accuracy of Different Mismatch Negativity Amplitude Representations in Predicting the Levels of Consciousness in Patients with Disorders of Consciousness

Kang Zhang[1], Kexin Li[3], ChunyunZhang[6], Xiaodong Li[4], Shuai Han[1], Chuanxiang Lv[1], Jingwei Xie[2], Li Bie[1*], Yongkun Guo[2,5*]

[1] Department of Neurosurgery, The First Hospital of Jilin University, Changchun, China

[2] Department of Neurosurgery, Fifth Affiliated Hospital of Zhengzhou University, Zhengzhou, China

[3] Department of Endocrinology, Jilin Province People's Hospital, Changchun, China

[4] Department of Neurosurgery, Siping Central People's Hospital, Siping, China

[5] Henan Key Laboratory of Brain Science and Brain Computer Interface Technology, Zhengzhou, China

[6] Department of Neurosurgery, Qilu Hospital of Shandong University(Qingdao), Qingdao, China

*** Correspondence:**

Li Bie, Department of Neurosurgery, The First Hospital of Jilin University, No.1 Xinmin Street, Changchun, Jilin Province ,130021, China. E-mail: bie_li@jlu.edu.cn

Yongkun Guo, Department of Neurosurgery, The Fifth Affiliated Hospital of Zhengzhou University, Zhengzhou; Henan Engineering Research Center for Prevention and Treatment of

Brain Injury, 3 Kangfuqian Street, Erqi District, Zhengzhou, Henan 450052, China. E-mail:

yongkunhope@126.com

Keywords: disorders of consciousness, mismatch negativity, microstate, functional connectivity, accuracy

Supplementary Table 1. Results of statistical testing for the duration of four microstates between conditions S and D.

Mean(SD)	D	S	P-value
**MS1**	86.71(15.50)	71.16(7.95)	0.10
**MS2**	16.93(10.75)	10.45(5.29)	0.15
**MS3**	7.49(4.72)	7.34(5.09)	1.00
**MS4**	19.34(4.30)	13.04(6.66)	0.18

MS1, MS2, MS3, and MS4 refer to four microstates respectively; D and S refer to condition D and condition S, respectively; SD, standard deviation.

Supplementary Table 2. Results of statistical testing for the duration of four microstates between MCS and VS groups.

Mean(SD)	MCS	VS	P-value
**MS1**	65.43 (14.25)	63.91(15.35)	0.87
**MS2**	14.05(8.62)	13.28(8.02)	0.93
**MS3**	9.16 (5.85)	5.83 (4.90)	0.20
**MS4**	16.68(13.45)	15.66(11.32)	0.99

MS1, MS2, MS3, and MS4 refer to four microstates respectively; MCS, minimally conscious state; VS, vegetative state; SD, standard deviation.
